# Phosphorylation of tau at Y18, but not tau-fyn binding, is required for tau to modulate NMDA receptor-dependent excitotoxicity in primary neuronal culture

**DOI:** 10.1186/s13024-017-0176-x

**Published:** 2017-05-19

**Authors:** Takashi Miyamoto, Liana Stein, Reuben Thomas, Biljana Djukic, Praveen Taneja, Joseph Knox, Keith Vossel, Lennart Mucke

**Affiliations:** 1 Gladstone Institute of Neurological Disease, 1650 Owens Street, San Francisco, CA 94158 USA; 2 Department of Neurology, University of California, San Francisco, San Francisco, CA 94158 USA; 3 Gladstone Institutes, Convergence Zone, 1650 Owens Street, San Francisco, CA 94158 USA

**Keywords:** Calcium, Excitotoxicity, Fyn, NMDARs, Phosphorylation, Tau, Y18

## Abstract

**Background:**

Hyperexcitability of neuronal networks can lead to excessive release of the excitatory neurotransmitter glutamate, which in turn can cause neuronal damage by overactivating NMDA-type glutamate receptors and related signaling pathways. This process (excitotoxicity) has been implicated in the pathogenesis of many neurological conditions, ranging from childhood epilepsies to stroke and neurodegenerative disorders such as Alzheimer’s disease (AD). Reducing neuronal levels of the microtubule-associated protein tau counteracts network hyperexcitability of diverse causes, but whether this strategy can also diminish downstream excitotoxicity is less clear.

**Methods:**

We established a cell-based assay to quantify excitotoxicity in primary cultures of mouse hippocampal neurons and investigated the role of tau in exicitotoxicity by modulating neuronal tau expression through genetic ablation or transduction with lentiviral vectors expressing anti-tau shRNA or constructs encoding wildtype versus mutant mouse tau.

**Results:**

We demonstrate that shRNA-mediated knockdown of tau reduces glutamate-induced, NMDA receptor-dependent Ca^2+^ influx and neurotoxicity in neurons from wildtype mice. Conversely, expression of wildtype mouse tau enhances Ca^2+^ influx and excitotoxicity in tau-deficient (*Mapt* ^−/−^) neurons. Reconstituting tau expression in *Mapt* ^−/−^ neurons with mutant forms of tau reveals that the tau-related enhancement of Ca^2+^ influx and excitotoxicity depend on the phosphorylation of tau at tyrosine 18 (pY18), which is mediated by the tyrosine kinase Fyn. These effects are most evident at pathologically elevated concentrations of glutamate, do not involve GluN2B–containing NMDA receptors, and do not require binding of Fyn to tau’s major interacting PxxP motif or of tau to microtubules.

**Conclusions:**

Although tau has been implicated in diverse neurological diseases, its most pathogenic forms remain to be defined. Our study suggests that reducing the formation or level of pY18-tau can counteract excitotoxicity by diminishing NMDA receptor-dependent Ca^2+^ influx.

**Electronic supplementary material:**

The online version of this article (doi:10.1186/s13024-017-0176-x) contains supplementary material, which is available to authorized users.

## Background

Experimental reduction of the microtubule-associated protein tau counteracts neural network hyperexcitability of diverse causes, including in animal models of severe childhood epilepsy and aging-related neurodegenerative disorders such as Alzheimer’s disease [1–7]. Conversely, neuronal overexpression of tau promotes network hyperexcitability [8–10]. Network hyperexcitability can lead to excessive release of excitatory neurotransmitters such as glutamate, which can cause neuronal injury and degeneration by overactivating NMDA-type glutamate receptors (NMDARs) and related signaling pathways (excitotoxicity) [11, 12]. Thus, excitotoxicity may be a key mechanism linking hyperexcitability and tau to neurodegeneration.

Multiple lines of evidence support the notion that tau plays a critical role in NMDAR-dependent excitotoxicity. For example, overexpression of human tau caused excitotoxicity in neuronal cultures [13]. In turn, glutamate-induced excitotoxicity increased tau expression [14, 15] and phosphorylation [16]. However, whereas the ability of tau reduction to suppress network hyperexcitability has been confirmed by multiple groups (see above), the potential of this strategy to suppress excitotoxicity has received less attention. Pizzi et al. [17] reported that pre-treating cultures of cerebellar granule cells with an anti-tau antisense oligonucleotide (ASO) for 1 h before exposing them to high concentrations of glutamate for 15 min reduced cell loss measured 24 h later. In light of the long half-life of tau [18–20], it is not surprising that this brief ASO treatment did not decrease baseline tau levels. Although the ASO did prevent acute, glutamate-induced increases in tau immunostaining, it is possible that its excitoprotective effect resulted from off-target effects. Furthermore, tau reduction in primary neurons did not affect kainate-induced calcium influx [7], another cause of excitotoxicity. Thus, more work is needed to define the roles of tau and tau reduction in excitotoxicity.

Although the exact mechanisms by which tau reduction counteracts network hyperexcitability, and possibly also downstream excitotoxicity, remain to be defined, several lines of evidence suggest that these mechanisms may involve Fyn. Fyn is a member of the Src family of tyrosine kinases [21]. It can phosphorylate tau at its tyrosine 18 residue to generate pY18-tau [22] and can bind to tau through one or more proline-rich (PxxP) motifs in tau [23–27]. Fyn can also modulate the phenotype of transgenic mice overexpressing human amyloid precursor proteins (hAPP) and hAPP-derived amyloid-β (Aβ) peptides in neurons. Like humans with AD [28, 29], these mice show evidence for network hyperexcitability, and suppression of this network hyperexcitability by tau reduction or treatment with the antiepileptic drug levetiracetam prevents or ameliorates synaptic and behavioral abnormalities in these models [1, 2, 30]. Neuronal overexpression of Fyn exacerbates network hyperexcitability and behavioral abnormalities in hAPP mice [31], whereas Fyn ablation reduces their synaptic impairments [32, 33].

Fyn phosphorylates the NMDAR subunit GluN2B at Y1472 [34, 35], which strengthens the interaction between NMDARs and PSD-95 in the postsynaptic density (PSD) [36] and enhances the activity of GluN2B–containing NMDARs [35]. Genetic ablation of tau in *Mapt* ^−/−^ mice has been reported to decrease the levels of Fyn in neuronal dendrites and synaptosome preparations, and to reduce the level of GluN2B phosphorylation at Y1472 [3]. These and other findings led Ittner et al. to hypothesize that tau enables interactions between Fyn and GluN2B through a process that involves binding of tau to Fyn and that tau reduction suppresses excitotoxicity by preventing Fyn from gaining access to GluN2B [3]. However, consistent with results obtained by others [2, 37], their electrophysiological recordings in acute hippocampal slices revealed that tau ablation did not change excitatory postsynaptic currents (EPSCs) or AMPA/NMDA current ratios, which seems at odds with the proposed hypothesis [3].

Here we used a primary neuronal culture model to further investigate the roles of tau and tau-Fyn interactions in glutamate-induced excitotoxicity, as well as the extent to which tau reduction can prevent this type of neurotoxicity, which has been implicated in a broad range of neurological diseases [11, 12].

## Results

To determine if tau has a role in excitotoxicity, we first established a potentially scalable cell-based assay to quantify excitotoxicity in cultures of primary mouse hippocampal neurons (hereafter referred to simply as “neurons”). For this purpose, we treated neurons with increasing concentrations of glutamate for 15 min, removed the glutamate, and measured reductions in mitochondrial activity 24 h later by alamarBlue assay as a surrogate marker of excitotoxic neuronal injury. Such short-term glutamate treatments are commonly used to study neurotoxicity [38–41] and could be relevant to the timeframe during which elevations in glutamate levels occur under pathological conditions, for example, as a result of surges in Aβ levels [42–45] or during seizures [46].

The alamarBlue assay uses resazurin, an oxidation-reduction indicator [47], to determine mitochondrial activity, which is altered by exposure of neurons to excitotoxic doses of glutamate [48, 49]. AlamarBlue fluorescence is more sensitive than other indicators of cytotoxicity [50–52] and, in neuronal cultures, changes linearly with cell number and decreases in response to toxic concentrations of excitatory amino acids, hypoxia, glucose deprivation, and abnormally low extracellular potassium levels [53]. Because glutamate dose is a critical determinant of excitotoxicity [12], we ensured that the assay covered a broad range of glutamate concentrations (0–500 μM).

**Fig. 1:**
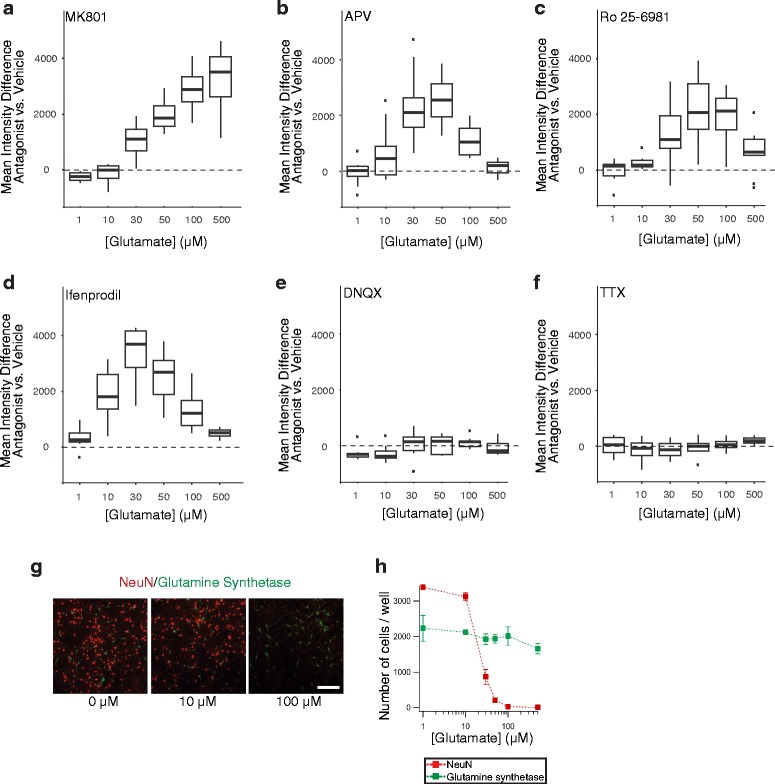
Glutamate-induced neurotoxicity depends on NMDARs. **a-f** Neurons from wildtype (WT) mice were exposed to different concentrations of glutamate for 15 min at DIV13. Relative levels of survival were quantified by alamarBlue assay 24 h later. Cultures were treated with vehicle or different antagonists starting 1 h before the glutamate exposure: **a** non-competitive NMDAR antagonist MK801 (20 μM), **b** competitive NMDAR antagonist APV (50 μM), non-competitive GluN2B-selective NMDAR antagonists **c** Ro 25–6981 (1 μM) or **d** ifenprodil (10 μM), **e** competitive AMPAR antagonist DNQX (20 μM), or **f** sodium channel blocker tetrodotoxin (TTX, 1 μM). The *boxplots *represent the distribution of the differences in mean fluorescence between antagonist- vs. vehicle-treated neurons at each dose across independent experiments. The *lower* and *upper ends* of the boxes represent the 25th and 75th quartile of each distribution, respectively. The *horizontal line* in each box represents the median. The *ends of the whiskers* terminate at the farthest points that are within 1.5 times the inter-quartile range (difference between upper and lower ends of the box). *Individual dots* shown in some of the panels represent outliers that fell outside the range defined by the whiskers. Consequently, the *curve* in **a** indicates that the survival-promoting effects of MK801 became more and more evident as glutamate concentrations increased, whereas the *bell-shaped curves* in **b**–**d** indicate that, at the particular concentrations used, the respective antagonists were protective at moderate, but not higher, concentrations of glutamate. Numbers of independent experiments (n) with cumulative well numbers per condition in parentheses: **a** 4 (24–32), **b** 10 (76–80), **c** 9 (68–72), **d** 8 (56–64), **e** 9 (70–72), and **f** 4 (30–32). When comparing mean differences across all doses within any given panel, a one-sided, one-sample t-test revealed significant differences between experimental and control conditions in (**a**, *P* < 0.05), (**b**, *P* < 0.0001), (**c**, *P* < 0.001), and (**d**, *P* < 0.001). **g, h** Neuronal cultures were treated with glutamate at DIV13 and fixed 24 h later, followed by immunostaining for the neuronal marker NeuN and the astroglial marker glutamine synthetase and nuclear staining with Hoechst33342. **g** Representative photomicrographs of immunolabeled cultures imaged on an ArrayScan XTI Live High Content Platform. Scale bar: 200 μm. **h** Number of Hoechst33342-positive cells per well that were immunoreactive for NeuN or glutamine synthetase. *n* = 4 independent experiments, each of which included 8–16 wells per condition. Data in **h** are means ± SEM

As expected [12, 54], glutamate exposure resulted in dose-dependent neurotoxicity (Fig. 1). Consistent with the prominent role of NMDARs in excitotoxicity [12] and the mode of action of their antagonists [55–59], the non-competitive NMDAR antagonist MK801 blocked most of the neurotoxicity over a wide range of glutamate concentrations (Fig. 1a), whereas the competitive NMDAR antagonist APV was more effective at moderate than high concentrations of glutamate (Fig. 1b). To assess the relative contribution of GluN2B-containing NMDARs, which have been implicated in excitotoxicity [12, 54], we applied Ro 25–6981 or ifenprodil, two non-competitive antagonists selective for such receptors [60, 61]. These antagonists also were only partly effective (Fig. 1c, d), consistent with other findings suggesting that GluN2B–containing NMDARs contribute to excitotoxicity more markedly at moderate than high doses of NMDAR agonists [54]. DNQX, a competitive antagonist of AMPA receptors (AMPARs), and tetrodotoxin (TTX), a sodium channel blocker, did not significantly attenuate neurotoxicity in our assay (Fig. 1e, f), although both drugs were bioactive (Additional file 1: Figure S1). Depletion of extracellular Ca^2+^ also shifted the dose-response curve to the right (Additional file 2: Figure S2A), consistent with the dependence of excitotoxicity on Ca^2+^ influx through NMDARs [12]. These findings agree with those of others indicating that AMPARs and neuronal activity are not required for excitotoxicity to occur [49, 62].

In line with the predominant role of NMDARs in excitotoxicity, the selective NMDAR agonist NMDA was as neurotoxic as glutamate (Additional file 2: Figure S2B). Although blocking AMPARs did not diminish glutamate-induced neurotoxicity (Fig. 1e), two AMPAR agonists, AMPA and L-quisqualic acid, caused intermediate levels of neurotoxicity in our assay (Additional file 2: Figure S2B). In contrast, the kainate receptor (KAR) agonist kainic acid elicited neurotoxicity only at very high doses, and the selective group I mGluR agonist (S)-3,5-DHPG had minimal effects (Additional file 2: Figure S2B).

To evaluate the relation of our neurotoxicity measurements to cell loss, we treated primary neuronal cultures with glutamate as above, confirmed glutamate-induced neurotoxicity by alamarBlue assay 24 h later, fixed and immunostained the cultures for the neuronal marker NeuN [63] and the astroglial marker glutamine synthetase [64], and counted the number of immunoreactive cells. Glutamate concentrations between 10 and 100 μM resulted in dose-dependent loss of neurons, but not astrocytes (Fig. 1g, h). In all subsequent experiments, we used the alamarBlue assay to evaluate neurotoxicity because it facilitates the quantitative analysis of multiple neuronal cultures and conditions.

**Fig. 2:**
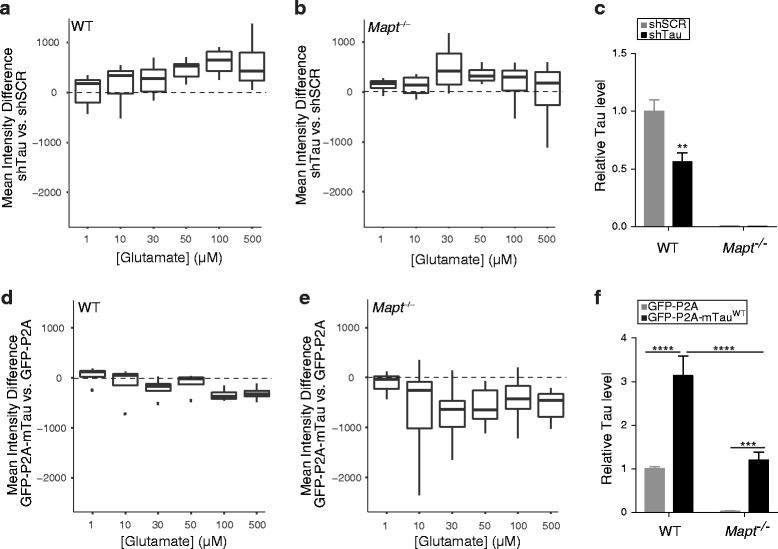
Tau modulates glutamate-induced excitotoxicity in cultured neurons. Glutamate-induced neurotoxicity in neuronal cultures from WT or tau-deficient (*Mapt* ^−/−^) mice was assessed as in Fig. 1. Some cultures were transduced with lentiviral vectors encoding shRNA against tau (shTau) or scrambled shRNA (shSCR) at the day of plating or with lentiviral vectors encoding GFP-P2A-mTau^WT^ or GFP-P2A at DIV7, as indicated. **a**, **b** Expression of shTau improved survival in WT **a** but not *Mapt* ^−/−^ **b** cultures. **c** Relative tau levels in WT and *Mapt* ^−/−^ cultures expressing shSCR or shTau were determined by western blot analysis with the pan-tau antibody EP2456Y and densitometric analysis of tau signals. Mean tau levels in shSCR-expressing WT cultures were arbitrarily defined as 1.0. **d**, **e** Overexpression of mTau^WT^ increased glutamate-induced neurotoxicity (i.e., decreased survival) in WT **d** and *Mapt* ^−/−^ **e** cultures. **f** Relative tau levels in WT and *Mapt* ^−/−^ cultures expressing GFP-P2A or GFP-P2A-mTau^WT^ were determined as in **c**. Mean tau levels in GFP-P2A-expressing WT cultures were arbitrarily defined as 1.0. Numbers of independent experiments (n) with cumulative well numbers per condition in parentheses: **a** 7 (42–56), **b** 4 (21–32), **c** 4–7 (1), **d** 4 (30–32), **e** 15 (106–120), and **f** 4–15 (1). When comparing mean differences across all doses within any given panel, a one-sided, one-sample t-test revealed significant differences between experimental and control conditions in (**a**, *P* < 0.01) and (**e**, *P* < 0.0001). **P < 0.01, ***P < 0.001, ****P < 0.0001 vs. control within same genotype by two-way ANOVA and Tukey test. Boxplots were explained in Fig. 1. Data in **c**, **f** are means ± SEM

Reduction of tau through lentiviral expression of anti-tau shRNA (shTau) significantly reduced glutamate-induced toxicity in neurons from wildtype but not tau-deficient (*Mapt* ^−/−^) mice (Fig. 2a-c, Additional file 3: Figure S3), particularly in the glutamate dose range in which we had observed neuronal loss in uninfected wildtype cultures (Fig. 1g, h). Knockdown with shTau reduced endogenous mouse tau (mTau) by ~50% (Fig. 2c). Conversely, lentiviral expression of wildtype 0N4R mTau (mTau^WT^) exacerbated glutamate-induced neurotoxicity in *Mapt* ^−/−^ but not wildtype neurons (Fig. 2d-f). These results suggest that tau contributes to excitotoxicity, especially in the presence of high glutamate concentrations.

**Fig. 3:**
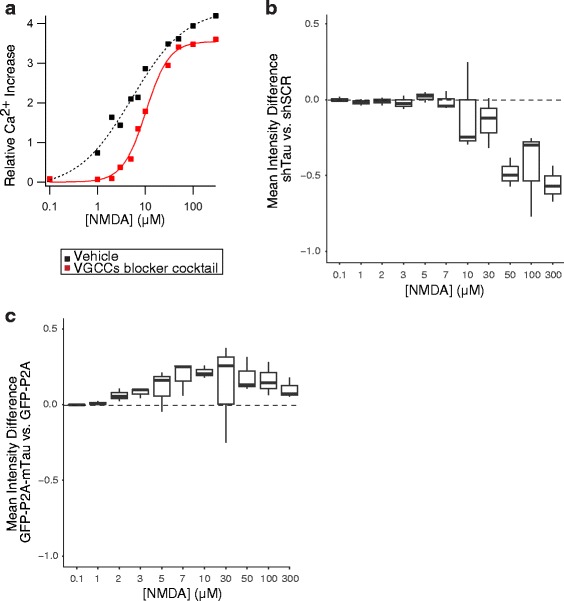
Tau modulates NMDAR-dependent Ca^2+^ influx. Live Ca^2+^ imaging was used to determine intracellular Ca^2+^ levels in WT neurons that were treated with different concentrations of NMDA at DIV14. **a** Representative relationship between NMDA doses and Ca^2+^ influx in neurons treated with vehicle or with a cocktail of voltage-gated calcium channel (VGCC) blockers for 30 min prior to and throughout NMDA application (see Methods). The VGCC blocker cocktail isolates NMDAR-dependent Ca^2+^ influx. Increases in fluorescence signals are expressed relative to baseline measurements obtained in the same wells. **b**, **c** In neurons treated with the VGCC blocker cocktail, tau knockdown decreased **b**, whereas tau overexpression increased **c** NMDAR-dependent Ca^2+^ influx. The boxplots represent the distribution of the differences in mean fluorescence between shTau- vs. shSCR-expressing neurons in **b** and GFP-P2A-mTau^WT^ vs. GFP-P2A expressing neurons in **c** at each dose across independent experiments. Numbers of independent experiments (n) with cumulative well numbers per condition in parentheses: **a** 1 (3), **b** 3 (9), and **c** 3 (9). When comparing mean differences across all doses within any given panel, a one-sided, one-sample t-test revealed significant differences between experimental and control conditions in (**b**: *P* < 0.05) and (**c**: *P* < 0.05)

Because Ca^2+^ influx through NMDARs critically contributes to excitotoxicity (Fig. 1a, Additional file 2: Figure S2A, and [49, 62]), we wondered whether tau might promote this process. To assess this possibility, we measured intracellular Ca^2+^ levels by live-cell Ca^2+^ imaging in neurons. We exposed neurons to increasing concentrations of NMDA while blocking voltage-gated Ca^2+^ channels (VGCC) (Fig. 3a), the other major route of Ca^2+^ entry into neurons [65, 66]. In this paradigm, tau reduction decreased, whereas tau overexpression increased NMDAR-dependent Ca^2+^ influx (Fig. 3b, c). Collectively, these results suggest that tau contributes to excitotoxicity, at least in part, by enhancing the cell surface levels or conductance of NMDARs.

**Fig. 4:**
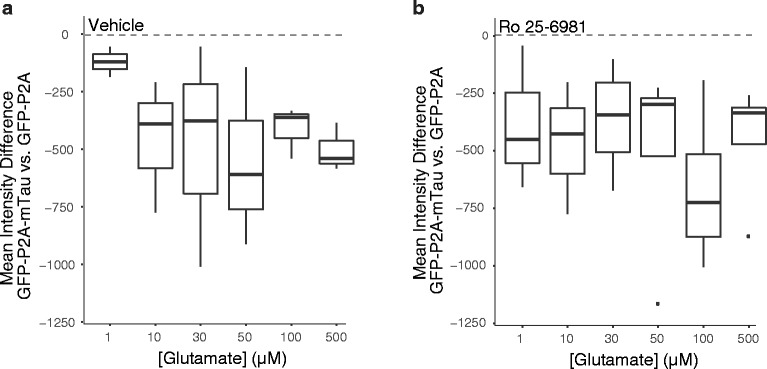
Enhancement of glutamate-induced excitotoxicity by tau does not depend on GluN2B/2B–containing NMDARs. **a**, **b** Glutamate-induced neurotoxicity in *Mapt* ^−/−^ neurons transduced with lentiviral vectors encoding GFP-P2A-mTau^WT^ or GFP-P2A was assessed as in Figs. 1 and 2. For 1 h before and throughout the glutamate exposure, neurons were treated with **a** vehicle or **b** Ro 25–6981 (1 μM). Numbers of independent experiments (n) with cumulative well numbers per condition in parentheses: **a** 3 (23–24) and **b** 4 (28–32). When comparing mean differences across all doses within any given panel, a one-sided, one-sample t-test revealed significant differences between experimental and control conditions in (**a**: *P* < 0.05) and (**b**: *P* < 0.05)

How might tau affect NMDARs? Ittner and colleagues proposed that tau-dependent transport of the tyrosine kinase Fyn to the PSD and subsequent phosphorylation of GluN2B by Fyn may modulate neuronal excitability and mediate Aβ-induced neuronal dysfunction [3]. However, Ro 25–6981 did not block the exacerbation of excitotoxicity caused by expression of mTau^WT^ in *Mapt* ^−/−^ neurons (Fig. 4a, b), making it unlikely that the contribution of tau to excitotoxicity depends on GluN2B–containing NMDARs, at least in our cell culture model.

**Fig. 5:**
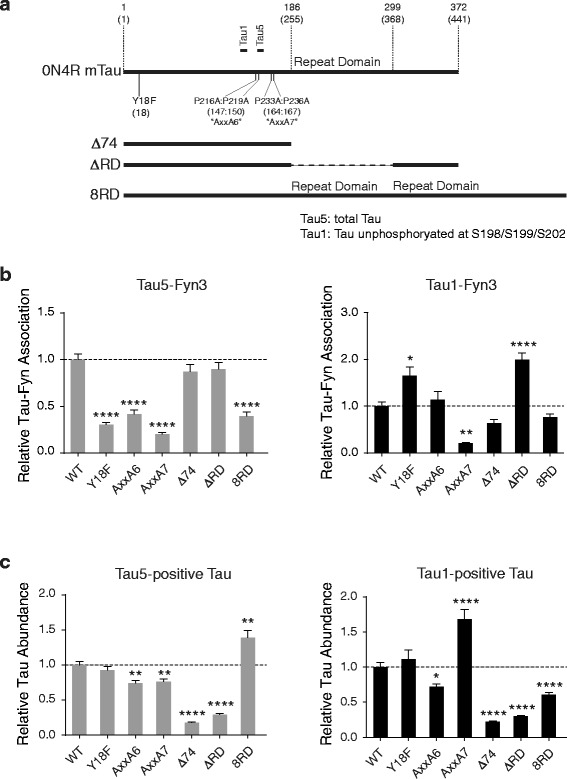
Tau mutations that modulate interactions between tau and Fyn. **a** Diagram indicating deletion and point mutations in 0N4R mTau and corresponding residues in 2N4R hTau in parentheses. Epitopes of the Tau1 and Tau5 antibodies are indicated also. **b** Interactions between Fyn and exogenous tau in transfected *Mapt* ^−/−^ neurons were determined by proximity ligation assay (PLA) using the Fyn3 antibody in combination with the Tau5 (*left*) or Tau1 (*right*) antibodies. Tau5 detects total tau, whereas Tau1 detects tau that is unphosphorylated at residues S198, S199 and S202. The closeness of interactions between Fyn and tau was expressed relative to measurements obtained in *Mapt* ^−/−^ neurons transfected with GFP-P2A-mTau^WT^ (WT). The other cultures were transfected with similar constructs in which mTau^WT^ was replaced by the indicated tau mutants. *n* = 16–31 neurons per construct from two experiments (three different neurites were quantified and averaged per individual transfected neuron). **c** The abundance of tau in transfected (GFP-positive) *Mapt* ^−/−^ neurons was estimated based on relative immunofluorescence intensities detected after staining of cultures with Tau5 (*left*) or Tau1 (*right*) and normalization to GFP signals. Average levels in GFP-P2A-mTau^WT^ (WT)-transfected cultures were arbitrarily defined as 1.0. *P < 0.05, **P < 0.01, ****P < 0.0001 vs. WT by one-way ANOVA and Bonferroni test. Data are means ± SEM

Notably, Fyn has multiple substrates and functions [21], raising the possibility that interactions between tau and Fyn [23–27] might contribute to Aβ-induced neuronal dysfunction [2, 31–33] and excitotoxicity in ways that do not depend on the phosphorylation of GluN2B by Fyn. To test this hypothesis, we constructed several viral vectors that encode 0N4R mTau bearing distinct mutations in putative Fyn interaction domains (Fig. 5a): substituting the single tyrosine residue that can be phosphorylated by Fyn [22] with phenylalanine (mTau^Y18F^), changing the prolines in each of two potential Fyn-binding PxxP motifs [24–27] to alanines (mTau^AxxA6^ and mTau^AxxA7^, respectively), and deleting both the repeat domain and the C-terminal domain (mTau^Δ74^). Overexpression of human Tau^Δ74^ (hTau^Δ74^) has been reported to sequester Fyn from the PSD and to reduce neuronal and cognitive dysfunction in hAPP transgenic mice [3]. To further assess the role of the repeat domain, which is critical for the binding of tau to microtubules [67], we also generated mTau constructs that lack this domain (mTau^ΔRD^) or have duplicate repeat domains (mTau^8RD^) [68, 69].

The interaction between tau and Fyn has been studied mostly in a cell-free system [24] and by co-immunoprecipitation [3, 23] using tagged hTau and an SH2 or SH3 fragment of Fyn [24–27]. To determine whether mTau interacts with endogenous Fyn in cultured neurons and assess how mutations in mTau affect their interaction, we used a proximity ligation assay (PLA), which enables in situ detection of two antigens in close proximity (<40 nm) (Additional file 4: Figure S4) [68, 70, 71]. In this assay, the antigens are labeled with primary antibodies, which are then bound by two secondary antibodies coupled with DNA strands that can ligate when in close proximity. The ligated sequence serves as an elongation template for quantitative PCR, whose product is labeled and detected by fluorescent probes [70]. Using this assay on *Mapt* ^−/−^ neuronal cultures transfected with the mTau constructs shown in Fig. 5a, we made the following observations. Mutation of Y18 or either PxxP motif reduced the association between total tau and Fyn, whereas deletion of the repeat domain—by itself or in conjunction with the C-terminal domain—did not (Fig. 5b). These findings provide further support for the importance of Y18 and both PxxP motifs in Fyn-tau interactions and suggest that these interactions do not require binding of tau to microtubules. In fact, because mTau^8RD^ bound more tightly to microtubules [68, 69] but less tightly to Fyn (Fig. 5b) than mTau^WT^, Fyn may interact primarily with tau that is not bound to microtubules and binding of tau to microtubules may diminish its chances of interacting with Fyn.

We also assessed the association of Fyn with tau that is unphosphorylated at residues S198, S199 and S202 (detected with the Tau1 antibody), an interaction that has remained controversial [23–27]. Mutation of Y18 and deletion of the repeat domain increased the association between this type of tau and Fyn, whereas the AxxA7 mutation decreased it, and no significant effects were observed as a result of the AxxA6 mutation, deletion of the repeat domain together with the C-terminal domain, or duplication of the repeat domain (Fig. 5b). These findings suggest that interactions between “unphosphorylated” tau and Fyn depend on only one of the two PxxP motifs and may be diminished by Y18 phosphorylation and binding of tau to microtubules.

All mTau constructs also encoded GFP, which was linked to mTau via a self-cleaving P2A peptide (GFP-P2A-mTau, Additional file 4: Figure S4A). This strategy can be expected to result in the production of mTau and GFP as separate proteins at a 1:1 molar ratio [72–75]. To control for differences in the expression of constructs across transfected neurons, we used GFP signals to normalize our measurements of mTau levels. Based on these measurements, we found that the AxxA6, Δ74 and ΔRD mutations decreased the relative levels of total and “unphosphorylated” tau, whereas the AxxA7 and 8RD modifications changed the relative abundance of these tau species in different directions (Fig. 5c). Reducing the binding of tau to microtubules (Δ74 and ΔRD) decreased tau levels, consistent with previous findings [3, 68, 69]. Interestingly, the AxxA7 mutation, which markedly reduced interactions of Fyn with both total and “unphosphorylated” tau (Fig. 5b), decreased the levels of total tau but prominently increased the levels of “unphosphorylated” tau (Fig. 5c). Underlying mechanisms may include changes in the stability, turnover or posttranslational modification of the respective tau species and deserve to be further explored.

**Fig. 6:**
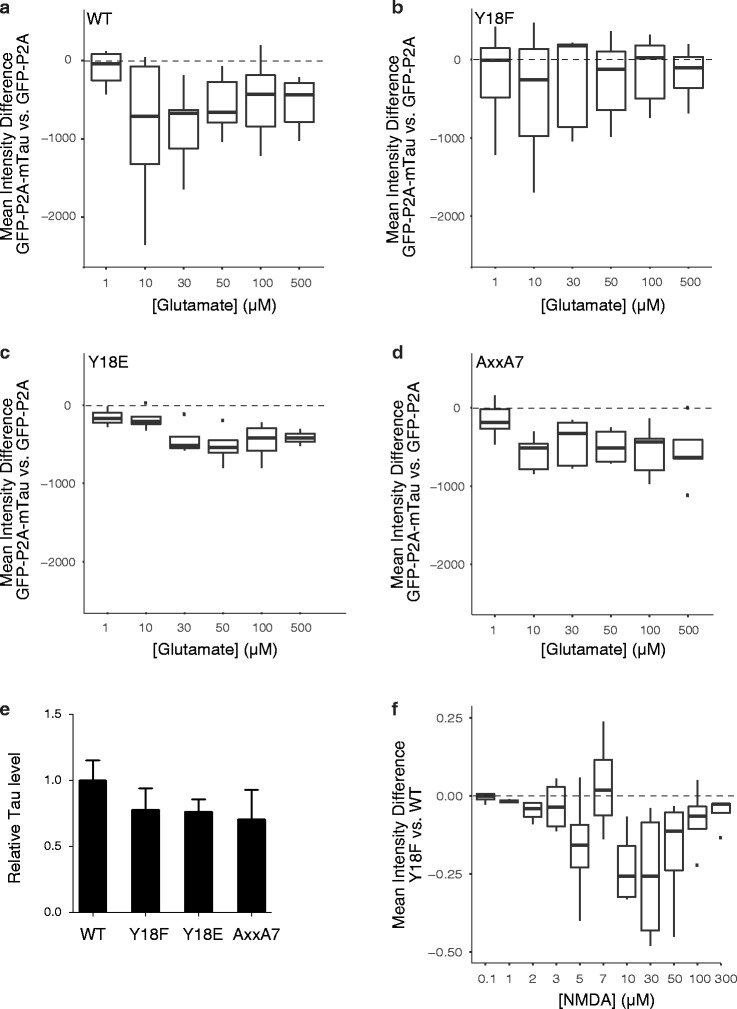
Phosphorylation of mTau at Y18 is required for tau to modulate glutamate-induced excitotoxicity. **a**-**d** Neurons from *Mapt* ^−/−^ mice were transduced with lentiviral vectors expressing GFP-P2A or GFP-P2A-mTau constructs encoding **a** mTau^WT^, **b** mTau^Y18F^, **c** mTau^Y18E^, or **d** mTau^AxxA7^ on DIV7. Glutamate-induced neurotoxicity was assessed as in Figs. 1 and 2. Numbers of independent experiments (n) with cumulative well numbers per condition in parentheses: **a** 10 (71–80), **b** 7 (47–56), **c** 4 (29–32), and **d** 5 (29–40). When comparing mean differences across all doses within any given panel, a one-sided, one-sample t-test revealed significant differences between experimental and control conditions in (**a**, *P* < 0.01), (**c**, *P* < 0.01) and (**d**, *P* < 0.01), but not (**b**, *P* = 0.12). **e** Relative tau levels in vehicle-treated cultures from a–d were determined by western blot analysis with the pan-tau antibody EP2456Y. Mean tau levels in GFP-P2A-mTau^WT^-expressing *Mapt* ^*−/−*^ cultures were arbitrarily defined as 1.0. One-way ANOVA revealed no significant differences in tau expression levels across cultures. Data are means ± SEM. **f** *Mapt* ^−/−^ neurons were transduced with lentiviruses encoding GFP-P2A-mTau^WT^ or GFP-P2A-mTau^Y18F^ on DIV7 and analyzed for NMDA-induced increases in intracellular Ca^2+^ in the presence of VGCC blocker cocktail on DIV 14 as in Fig. 3. Numbers of independent experiments (n) with cumulative well numbers per condition in parentheses: 4 (12). When comparing mean differences across all doses, a one-sided, one-sample t-test revealed significant differences between mTau^Y18F^ and mTau^WT^ (*P* < 0.01)

To determine whether tau-Fyn interactions are involved in the tau-dependent modulation of excitotoxicity, we focused on the Y18F mutation, which disrupts Fyn-dependent phosphorylation of tau at this residue [23] and the AxxA7 mutation, which interferes with the binding of tau to Fyn (Fig. 5b) [3, 24]. Neurons from *Mapt* ^−/−^ mice were transduced with lentiviral vectors expressing mTau^WT^, mTau^Y18F^, a phosphomimetic mutant at the Y18 residue (mTau^Y18E^), or mTau^AxxA7^, followed by exposure to glutamate and the neurotoxicity assay. At >30 μM of glutamate, mTau^WT^, mTau^Y18E^ and mTau^AxxA7^ similarly increased glutamate-induced neurotoxicity beyond levels in GFP-P2A-expressing tau-deficient neurons, whereas mTau^Y18F^ was significantly less effective (Fig. 6a-d). All of these mTau proteins were expressed at comparable levels (Fig. 6e). In *Mapt* ^−/−^ neurons exposed to NMDA in the presence of VGCC blockers, expression of mTau^Y18F^ increased NMDAR-dependent Ca^2+^ influx less than expression of mTau^WT^ (Fig. 6f).

## Discussion

These results suggest that tau promotes glutamate-induced excitotoxicity by increasing NMDAR-dependent Ca^2+^ influx and that Fyn-mediated phosphorylation of tau at Y18 enhances this process at high concentrations of glutamate. These effects did not involve GluN2B-containing NMDARs and did not require binding of Fyn to tau’s major interacting PxxP motif or of tau to microtubules. In addition to these novel findings, we confirmed that glutamate-induced excitotoxicity is inhibited by 50% reduction of tau [1, 3, 17] and exacerbated by overexpression of tau [9, 10].

We assessed excitotoxicity over a wide range of glutamate concentrations because it is difficult to know how glutamate concentrations in primary neuronal cultures relate to those in the intact or diseased brain. On one hand, the isolation of primary neurons from the brain environment exposes them to many stresses that could increase their susceptibility to excitotoxicity. On the other hand, their culture medium contains numerous neuroprotective factors that could diminish the neurotoxic impact of any given glutamate concentration. In the healthy brain, glutamate is the most abundant free amino acid, but its levels vary widely depending on the exact location. Intracellular glutamate concentrations in the brain are in the millimolar range [76]. The concentration of glutamate in the synaptic cleft ranges from 0.5 to 1.0 mM [77]. The extracellular concentration of glutamate is normally kept in the low micromolar range, for example, at 2 μM in the hippocampus [78], 9 μM in the cortex [76], and 10 μM in the CSF [79]. However, glutamate concentrations probably vary more widely in the complex pathological microenvironments of AD brains, the penumbra of an ischemic infarct, and brain regions affected by epileptic seizures. Compared with controls, glutamate levels were increased ~1.3-fold in the CSF of AD patients [80] and ~2-fold in the extracellular space of the hippocampus of mice overexpressing human APP, which simulate key aspects of AD [44]. Even greater increases in glutamate concentrations may occur in AD brains within and around synaptic clefts: soluble Aβ oligomers, which accumulate in AD brains [81–83], elicit glutamate release from astrocytes [44] and microglia [45], and prevent excitatory amino acid transporters from clearing extracellular glutamate by negatively affecting their expression or function [43, 84, 85]. Bilateral intrahippocampal microdialysis in humans revealed that extracellular glutamate concentrations can be in the 100–500 μM range for over 15 min during seizures [46]. The experimental conditions under which we observed protective effects of tau reduction may be relevant to some or all of these pathophysiological scenarios.

Indeed, the excitoprotective effects of tau reduction we observed in this study were most evident at pathologically elevated concentrations of glutamate. At lower concentrations of glutamate, tau reduction had no effect on multiple outcome measures, including NMDA-induced Ca^2+^ influx. These observations are consistent with in vivo studies demonstrating that reduction or ablation of tau has minimal or no effects on neuronal activities unless pathological conditions are introduced that cause network hyperexcitability [1–6, 86]. While these studies indicate that tau reduction counteracts network hyperexcitability of diverse causes, the current study suggests that tau reduction may also counteract the excitotoxic neuronal injury to which network hyperexcitability can lead, highlighting the broad potential of this therapeutic strategy.

High concentrations of glutamate are associated with an increased opening time of NMDARs [87], which may explain, at least in part, why the effects of tau modulation were most evident at high doses of glutamate. It is noteworthy in this context that our measurements may have underestimated tau-dependent changes in NMDAR-dependent Ca^2+^ influx. We selected FLIPR Calcium 6 (Kd = 320 nM) as an indicator because it has a high quantum yield as well as a broad signal window and amplitude. However, because this indicator can become saturated at high intracellular Ca^2+^ concentrations, it may not be optimal for detecting changes in Ca^2+^ influx at the high glutamate concentrations at which tau modulation has the most marked effects on excitotoxicity. Furthermore, our Ca^2+^ imaging assay measured overall cytosolic Ca^2+^ levels, whereas the effects of tau modulation on intracellular Ca^2+^ levels might be confined to specific subcellular regions that are particularly critical for downstream signaling such as the area near the mouth of NMDARs [88]. Although Ca^2+^ influx plays an important role in excitotoxicity [11, 12, 89], depletion of extracellular Ca^2+^ only partially protected against excitotoxicity in our model, possibly because we returned our neurons to Ca^2+^-containing medium right after the glutamate exposure. Ca^2+^-independent metabotropic functions of NMDA receptors could also contribute to cellular toxicity [90]. It should also be noted that glutamate-induced excitotoxicity may depend less on extracellular and cytoplasmic Ca^2+^ levels than on mitochondrial Ca^2+^ levels [11, 91–93], which we did not measure. Thus, the relationship between tau, Ca^2+^ homeostasis, and excitotoxicity is probably complex and deserves further investigation.

The mTau^WT^-dependent enhancement of glutamate-induced neurotoxicity in our neuronal cultures did not depend on GluN2B–containing NMDARs, as it was observed in the absence (Figs. 2, 4, and 6) or presence (Fig. 4) of Ro-25-6981. Because the NMDAR subunit composition in cultured neurons changes from containing more GluN2B to containing more GluN2A [94] and our DIV13 neuronal cultures may still be in this transition, we cannot exclude the possibility that this tau effect occurs in maturing, but not adult, neurons. However, DIV12–14 is a standard time point for measuring excitotoxicity [95–97], and the GluN2B-to-GluN2A subunit transition at this time point is largely completed [98, 99]. Moreover, treatment with Ro 25–6981 or ifenprodil attenuated glutamate-induced excitotoxicity in our cultures at moderate but not high doses of glutamate, consistent with results obtained in cortical neurons at DIV18 [54].

Because Tau^Y18F^ was unable to enhance excitotoxicity at high glutamate concentrations, a potential excitoprotective strategy may be to inhibit phosphorylation of tau by Fyn, a process that appears to be restricted to Y18 [22]. In principle, this could be achieved by inhibiting the kinase activity of Fyn. However, although several inhibitors of other members of the Src kinase family have been developed for the treatment of cancer [100] and one of these inhibitors is being tested in a clinical trial for AD [101, 102], we are unaware of drugs that selectively inhibit Fyn, but none of the other members of the Src kinase family, and that also effectively penetrate the blood-brain barrier. Furthermore, like the other members of this family, Fyn has diverse substrates and has been implicated in multiple pathways that affect neuronal functions [102–104]. Genetic ablation of Fyn in mice did not cause overt phenotypes but reduced calcium flux in thymocytes [105], impaired LTP in the olfactory bulb and hippocampus [106, 107], and caused spatial learning deficits [106]. Heterozygous Fyn knockout mice had no detectable behavioral or anatomical deficits [107], suggesting that partial reduction of Fyn may be well tolerated. Indeed, cognitive deficits and synaptic depletion in APP/PS1 mice were reversed by treatment with the Src family kinase inhibitor AZD0530, which inhibits Fyn and is well tolerated in mice, dogs, and humans [101, 102]. Other potential strategies include blocking protein-protein interactions that are required for Fyn to phosphorylate Y18 in tau, and targeting downstream consequences of this posttranslational modification.

The phosphomimetic Y18E substitution in mTau did not increase glutamate-induced neurotoxicity beyond levels observed with mTau^WT^. Although glutamate is commonly used to mimic tyrosine phosphorylation, it should be noted that glutamate and phosphorylated tyrosine show larger structural differences than other phosphomimetic substitutions (e.g., aspartate versus phosphorylated serine) [108], which may have contributed, at least in part, to the negative result we obtained with mTau^Y18E^.

Previous studies showed that phosphorylation of Y18 disrupts binding of hTau to Fyn’s SH3 domain [25] but is required for binding of hTau to Fyn’s SH2 domain [27]. In our PLA experiments, blocking Y18 phosphorylation of mTau by mutagenesis shifted the interaction of Fyn from “total” tau to “unphosphorylated” tau. By extrapolation, it is possible that Y18 phosphorylation promotes the interaction of Fyn with tau species that are also phosphorylated at residues S198, S199 and S202. Additional studies are needed to test this hypothesis and to characterize the intraneuronal distribution of pY18-tau. Other tau phosphorylations have been implicated in the enrichment of tau in dendritic spines and in consequent alterations of NMDARs [109–112]. In an extensive mass spectrometry analysis of posttranslational tau modifications, we did not detect pY18-tau in hippocampal and cortical homogenates from WT or hAPP transgenic mice [113], suggesting that this species was of low abundance or absent from these tissues. Because pY18-tau may be confined to a small subcellular compartment, its detection would likely require the development of sensitive pY18-tau-specific antibodies that are suitable for immunostaining of brain sections and electron microscopy; to our knowledge, such antibodies are currently not available. Closely related to the subcellular distribution of pY18-tau is the question of how this tau species might increase NMDAR-dependent Ca^2+^ influx. Possible mechanisms include interactions of tau with NMDARs, regulators of NMDARs or other component of the PSD, and the modulation of signaling pathways that can increase intracellular Ca^2+^ levels. Additional studies are needed to explore these nonexclusive possibilities.

## Conclusions

Our findings suggest that neuronal tau accumulation promotes and tau reduction counteracts glutamate-induced excitotoxicity, most likely by modulating NMDAR-dependent Ca^2+^ influx. The tau-dependent enhancement of excitotoxicity requires phosphorylation of tau at its Y18 residue, which is mediated by Fyn, but does not depend on interactions of Fyn with known Fyn-binding domains in tau or on GluN2B/2B–containing NMDARs. Blocking the phosphorylation of tau at Y18 may be excitoprotective, particularly in pathological conditions associated with an abnormal accumulation of tau, glutamate or both.

## Methods

### Reagents

Table 1 provides information on the antibodies and Table 2 on the pharmacological compounds used in this study.

**Table 1:** Antibodies used for western blotting (WB), immunocytochemistry (ICC), or proximity ligation assay (PLA)

Antibody/Clone	Target	Source	Final Concentration/ Dilution	Method
AHB0042 (clone: Tau5)	Tau	Thermo Fisher Scientific	1 μg/ml	WB, ICC, PLA
MAB10417 (clone: EP2456Y)	Tau	EMD Millipore	0.1 μg/ml	WB
MAB302 (clone: GS-6)	Glutamine Synthetase	EMD Millipore	1:1000	ICC
MAB3420 (clone: Tau1-PC1C6)	Tau	EMD Millipore	1 μg/ml	WB, ICC, PLA
MAB377 (clone: A60)	NeuN	EMD Millipore	1:1000	ICC
sc-16 (clone: Fyn3)	Fyn	Santa Cruz Biotechnology	1 μg/ml 4 μg/ml	WB, PLA
T5076-200UL (clone: SDL.3D10)	βIII Tubulin	Sigma-Aldrich	0.1 μg/ml	WB
Secondary antibodies conjugated with Alexa Fluor	Respective species and isotype	Thermo Fisher Scientific	2 μg/ml	ICC
Secondary antibodies conjugated with IRDye	Respective species and isotype	LI-COR	0.1 μg/ml	WB

**Table 2:** Compounds (all from Tocris Biosciences)

Compound	Catalog Number	Solvent of Stock
(+)-MK 801 maleate	0924	Water
AMPA	0169	Water
DL-APV	0105	Water
DNQX	0189	DMSO
Ifenprodil	0545	Water
Kainic acid	0222	Water
L-Quisqualic acid	0188	1 eq. NaOH
Mibefradil	2198	Water
Nimodipine	0600	DMSO
NMDA	0114	Water
Ro 25–6981 maleate	1594	DMSO
(S)-3,5-DHPG	0805	Water
SNX 482	2945	Water
TTX	1078	Water
ω-agatoxin IVA	2799	Water
ω-conotoxin MVIIC	1084	Water

### Primary mouse hippocampal cultures

For the generation of primary cultures, we used male and female WT mice and *Mapt* ^−/−^ mice [114] (kindly provided by Dr. Hana Dawson, Duke University) on a C57Bl/6 J background. Hippocampi of newborn pups (P0–P1) were dissected in ice-cold Earle’s balanced salt solution (EBSS), lacking CaCl_2_, MgSO_4_ and phenol red (Thermo Fisher Scientific, 14155), digested with papain (Worthington LK003176, ~1 unit per hippocampus) in EBSS at 37 °C for 15 min, and triturated in a disposable plastic tube in low ovomucoid solution: Dulbecco’s phosphate-buffered saline (DPBS, Thermo Fisher Scientific, 14040-182) containing 1.5 mg/ml BSA (Sigma-Aldrich, A7030-10G), 1.5 mg/ml trypsin inhibitor (Sigma-Aldrich, T9253-5G), and 66.7 units/ml DNase I (Sigma-Aldrich, D5025). After removing debris with a 70-μm nylon strainer (BD Biosciences, 352350), neurons were spun at 1000 rpm for 5 min. Cell pellets were gently dissociated in Neurobasal A medium supplemented with 1× B27 (Thermo Fisher Scientific, 17504–044), 1× N2 (Thermo Fisher Scientific, 17502–048), 2.4 mM L-glutamine (Thermo Fisher Scientific, 25030–081), and 100 units/ml penicillin/streptomycin (Thermo Fisher Scientific, 15410–122). Cells were plated on poly-D-lysine (PDL)-coated 12-well or 96-well plates at a density of 500,000 or 60,000 neurons/well, respectively, for western blot analyses or glutamate-induced excitotoxicity assay, or 800,000 neurons/coverslip for proximity ligation assays. Half of the medium was replaced with new medium every week. Neurons were used for experiments at DIV 13–15. By DIV14, cultures typically contained similar numbers of neurons and astrocytes (Fig. 1a, h).

### Glutamate-induced excitotoxicity assay

Primary cells were plated on PDL-coated 96-well plates (Greiner Bio-one, 655946) at a density of 60,000 cells per well. Cultures were infected with lentiviral constructs encoding shRNA on the day of plating (DIV0) or with lentiviral constructs encoding GFP-P2A or GFP-P2A-mTau on DIV7. On DIV13, cells were treated with various concentrations of glutamate dissolved in Neurobasal A medium with supplements (1× B27, 1× N2, 2.4 mM L-glutamine, and 100 units/ml penicillin/streptomycin) or mock treated with Neurobasal A medium with supplements for 15 min at 37 °C under 5% CO_2_, followed by incubation conditioned medium previously collected from the same cultures. For Additional file 2: Figure S2A, cells were exposed to glutamate in either Ca^2+^-containing isotonic solution (vehicle [all in mM]: 138 NaCl, 5 KCl, 1 MgCl_2_, 2 CaCl_2_, 10 HEPES, and 10 glucose) or in 0 mM Ca^2+^ solution (138 NaCl, 5 KCl, 1 MgCl_2_, 0.1 EGTA, 10 HEPES, and 10 glucose). At 24 h after glutamate treatment, conditioned medium was replaced with pre-warmed HEPES-buffered solution (HBS [all in mM]: 119 NaCl, 2.5 KCl, 2 MgCl_2_, 2 CaCl_2_, 25 HEPES, and 30 glucose) containing 1× alamarBlue (Thermo Fisher Scientific, DAL1100), and cultures were incubated at 37 °C under 5% CO_2_ for 2–3 h. AlamarBlue fluorescence was then measured with a fluorescence plate reader (Molecular Device, SpectraMax M5). Mock treated cells were homogenized in RIPA lysis and extraction buffer (Thermo Fisher Scientific, 89900) with Halt protease and phosphatase inhibitor cocktail (Thermo Fisher Scientific, 78447) to assess tau levels by western blot analysis.

### High content imaging

After the excitotoxicity assay, cultures were fixed with 4% paraformaldehyde and 4% sucrose in 1× phosphate buffered saline (PBS) for 15 min at room temperature (RT) and washed three times with 1× PBS. Fixed cells were permeabilized with 0.2% Triton X-100 in 1× PBS for 5 min and blocked with 10% normal goat serum (NGS, Jackson Immunoresearch, 005–000–121) in 1× PBS for 1 h at RT. They were then incubated with primary antibodies against the neuron-specific marker NeuN (Millipore, MAB377) and the glia-specific marker glutamine synthetase (GS, Millipore, MAB302) in 1× PBS containing 2% NGS for 1.5 h at RT. Cultures were then washed three times with 1× PBS at RT and stained with Hoechst33342 (1:5000 dilution, Thermo Fisher Scientific, H3570) to visualize nuclei and with secondary antibodies conjugated with Alexa Fluor546 (A-21123) for NeuN or Alexa Fluor647 (A-21241) for GS (2 μg/ml, Thermo Fisher Scientific) in 1× PBS containing 2% NGS for 1 h at RT. Cultures were then washed three times with 1× PBS and imaged on an ArrayScan XTI Live High Content Platform (Thermo Fisher Scientific). Twenty-one images were taken per well of a 96-well plate using a 10× objective. Filter settings were 549–15 BGRFRN for Alexa Fluor546, 650–13 BGRFRN for Alexa Fluor647, and 386–23 BGRFRN for Hoechst33342. Exposure time for each channel was determined with the built-in peak target (percentile) method, so that target values were between 20% and 25%. After acquiring all images, the number of punctae that were positive for Hoechst33342 and NeuN or GS were counted using the colocalization program of HCS Studio: Cellomics Scan Version 6.5.0 (Thermo Fisher Scientific). Hoechst33342-positive punctae were determined using the following parameters. Smoothing: Uniform (Method: Value = 1), Thresholding: Isodata (Method: Value = −0.25), Segmentation: Shape (Method: Value = 1), and Object cleanup option on. Pre-defined Hoechst33342-positive punctae that were also positive for NeuN or GS were determined using primary object mask modification (Mask type: Circle and Value = 1) and the object selection option by defining the smallest minima in the histograms of both Object.TotalIntensity and Object.AvgIntensity as the low object selection levels and the highest values as the high object selection levels.

### Live Ca^2+^ imaging with Flexstation3

Neurons were plated on PDL-coated 96-well plates (Greiner Bio-one, 655946) at a density of 80,000–120,000 neurons per well, and transduced with lentiviral vectors encoding shRNA on the day of plating (DIV 0) or with lentiviral vectors encoding GFP-P2A or GFP-P2A-mTau at DIV7. At DIV14, neurons were incubated with fluorescent Ca^2+^ indicator (Molecular Devices, FLIPR Calcium 6 assay kits) in HBSS-Hepes buffer (1× Hank’s balanced salt solution, 20 mM HEPES, 2.5 mM probenecid, 30 mM glucose, and 50 μM glycine) for 1.5 h in the incubator (37 °C, 5% CO_2_). Voltage gated calcium channel (VGCC) blocker cocktail (ω-conotoxin MVIIC [0.5 μM], SNX482 [0.15 μM], ω -agatoxin IVA [0.1 μM], nimodipine [20 μM], and mibefradil [10 μM]) and Ro 25–6891 were added to some cultures 30 min before the imaging and not washed off. The fluorescence intensity of each well was monitored every 1.28 s for 60 s. HBSS-Hepes buffer containing various concentrations of NMDA was applied 16 s after the beginning of the recording. For each well, the maximal fluorescence increase (ΔF) was normalized to the baseline fluorescence (F_0_). The ΔF/F_0_ ratio of each well was divided by the average ΔF/F_0_ ratio of wells treated with ionomycin (1 μM).

### Western blot analysis

Equal amounts of total protein (15 μg per lane) in 1× NuPAGE LDS sample buffer (Thermo Fisher Scientific, NP0007) and 1× Sample Reducing Agent (Thermo Fisher Scientific, NP0009) were loaded per gel lane. Protein samples were electrophoresed on NuPAGE Novex 4–12% Bis-Tris Midi protein gels (Thermo Fisher Scientific, WT1403A) in 1X NuPAGE MOPS SDS running buffer (Thermo Fisher Scientific, NP0001–02) at 200 V for 1 h at RT. Gels were transferred to nitrocellulose membranes with an iBlot gel transfer device (Thermo Fisher Scientific). Membranes were blocked with Odyssey blocking buffer (LI-COR, 927–40000) for 1 h at RT, incubated with primary antibodies overnight at 4 °C, washed with TBS containing 0.05% Tween20 (TBST) four times for 5 min at RT, incubated with matching secondary antibodies conjugated to IRDye (LI-COR, 0.1 μg/mL) for 1 h at RT, and washed in TBST four times for 5 min at RT. Protein bands were visualized with an Odyssey CLx Infrared Imaging System (LI-COR) and quantified with Image Studio software (LI-COR).

### Generation of mTau mutants

A cDNA encoding WT 0N4R mTau was linked to a red-shifted variant of GFP (RSGFP4) [115] via a P2A linker that contained a furin cleavage site and a V5 tag [116] by PCR. The resulting GFP-P2A-mTau construct was inserted into a pFUW plasmid between AscI and RsrII sites. A short sequence of mTau 3′ UTR (Fragment H) was inserted into this plasmid between RsrII and HpaI sites. Fragment H is homologous to a 3′ UTR region of rat tau that stabilizes tau mRNA and promotes its axonal localization [117, 118]. The RsrII site between mTau and Fragment H was deleted using the Quikchange II XL site-directed mutagenesis kit (Agilent Technologies 200522) and the following primers: 5′-gatccggcgcgccatggtgagcaagg-3′ and 5′-ccttgctcaccatggcgcgccggatc-3′. We refer to the resulting plasmid as GFP-P2A-mTau^WT^. The GFP-P2A control plasmid lacks the mTau cDNA and has a tga stop codon at the 3′ end of the P2A linker. The Quikchange II XL site-directed mutagenesis kit was used to introduce mutations into GFP-P2A-mTau^WT^ using the primers listed in Table 3.

**Table 3:** Primers used

Y18F_s	5′- agaccatgctggagatttcactctgctccaagac -3’
Y18F_as	5′- gtcttggagcagagtgaaatctccagcatggtct -3’
Y18E_s	5′- gaagaccatgctggagatgagactctgctccaagacc -3’
Y18E_as	5′- ggtcttggagcagagtctcatctccagcatggtcttc -3’
AxxA6_s	5′- accccatccctagcaacaccggccacccgg -3’
AxxA6_as	5′- ccgggtggccggtgttgctagggatggggt -3’
AxxA7_s	5′- ggtccgcactcccgctaagtcagcatcagctagtaa -3’
AxxA7_as	5′- ttactagctgatgctgacttagcgggagtgcggacc -3’

After mutagenesis, plasmids were validated by sequence analysis and used to transform the Stbl3 *E. coli* strain (Thermo Fisher Scientific, C7373–03) for maintenance. The constructs encoding mTau^noRD^ or mTau^8RD^ were described previously [68].

### Production and purification of lentiviral particles

Lentiviral particles were generated by co-transfecting the transfer vector (pFUGW containing shSCR or shTau, and pFUW containing GFP-P2A, GFP-P2A-mTau^WT^, or GFP-P2A-mTau with mTau mutations), the HIV-1 packaging vector (Delta8.9), and the VSVG envelope glycoprotein expression vector (pVSVG) into HEK293T cells. Confluent HEK293T cells were transfected with 22.5 μg of transfer vector, 16.9 μg of Delta8.9, and 11.25 μg of pVSVG per 15 cm petri dish using CalPhos transfection reagent (Clontech, 631312) according to the manufacturer’s instructions. Medium containing lentiviral particles was collected 48 h after transfection and filtered through a 0.22-μm cellulose acetate filter (Corning Incorporated, 431154). Lentiviral particles in the medium were then concentrated by serial ultracentrifugation: 21,000 rpm for 2 h at 4 °C in a Beckman SW28 and then 25,000 rpm for 2 h at 4 °C in a Beckman SW55 with a sucrose cushion consisting of 2 ml of 20% sucrose in Hank’s balanced salt solution (HBSS, Thermo Fisher Scientific, 14170) at the bottom of the SW55 tubes. Final pellets were dissolved in HBSS, aliquoted, and stored at -80 °C until use. Lentiviral titers were determined with a p24 ELISA. Neuronal cultures were transduced with lentiviral particles encoding shRNA at 3 fg p24 per neuron on the day of plating (DIV0) or with lentiviral particles encoding mTau at 0.02 pg p24 per neuron on DIV7. Lentiviral vectors encoding shRNA against mTau were described previously [68]. Briefly, the target sequence for the anti-tau shRNA was 5′- acagagtccagtcgaagatt -3′. The shRNA was placed under control of the U6 promoter. The U6-shRNA expression cassette (pSilencer 2.0, Ambion) was inserted between the NheI and PacI sites of pFUGW plasmid, upstream of a ubiquitin C promoter directing expression of EGFP. A similar construct expressing an shRNA targeting a scrambled sequence (5′- ccactaccgttgttataggtg -3′) was used as a control.

### Proximity Ligation Assay (PLA)

On DIV 7, 10^6^ neurons from *Mapt* ^−/−^ P0–P1 pups were plated on 12-mm poly-D-lysine/laminin-coated glass coverslips (BD Biosciences, 354087), incubated in Neurobasal A medium containing kynurenic acid (1 mM, Sigma-Aldrich, K3375-5G) and GlutaMAX (0.5 mM, Thermo Fisher Scientific, 35050–061) at 37^o^ C with 5% CO_2_ for 30–60 min, and transfected with pFUW plasmids encoding GFP-P2A or GFP-P2A-mTau. To prepare the transfection mixture, each plasmid (1.0 μg per coverslip) was dissolved in 50 μl Opti-MEM (Thermo Fisher Scientific, 31985–062), mixed with 50 μl Opti-MEM containing 1.35 μl Lipofectamine 2000 (Thermo Fisher Scientific, 11668–027), and incubated for 20 min at RT. Neuronal cultures were incubated in the transfection mixture for 30 min at 37^o^ C with 5% CO_2_. Cultures were then washed with pre-warmed PBS once and placed back into conditioned medium collected from the same cultures before transfection. PLA was performed according to the protocol of Duolink In Situ/Fluorescence (Sigma-Aldrich) one day after transfection. Neurons were fixed with 4% paraformaldehyde/4% sucrose in 1× PBS for 15 min at RT, washed with 1× PBS (3 × 5 min), permeabilized with 0.2% Triton X-100 in 1× PBS for 5 min, and blocked with 5% normal goat serum (NGS, Jackson Immunoresearch, 005–000–121) in 1× PBS for 30 min at RT. They were then incubated with primary antibodies (Tau1: 1 μg/ml, Tau5: 2 μg/ml, Fyn3: 4 μg/ml) in 1% NGS/1× PBS at 4 °C overnight. After washing with 1× PBS (3 × 5 min), neurons were incubated with two PLA probes (Duolink In Situ PLA Probe Anti-Rabbit PLUS DUO92002 and Duolink In Situ PLA Probe Anti-Mouse MINUS DUO92004, Sigma-Aldrich) in 1% NGS/1× PBS for 1 h at 37 °C, followed by washes (2 × 5 min) with 1× Wash Buffer A (Sigma-Aldrich, DUO82049-4 L) and incubation in ligation solution (Sigma-Aldrich, Duolink In Situ Detection Reagents Red, DUO92008) for 1 h at 37 °C. After ligation of complementary PLA probes that were in close (<40 nm) proximity, neurons were washed with 1× Wash Buffer A (2 × 2 min) and incubated in the amplification solution (Sigma-Aldrich, Duolink In Situ Detection Reagents Red, DUO92008) for 100 min at 37 °C to fluorescently label the ligated PLA probes. Neurons were then serially washed in 1× Wash Buffer B (2 × 10 min, Sigma-Aldrich, DUO82049-4 L), 0.01× Wash Buffer B (1 × 1 min), and 1× PBS (1 × 5 min), followed by incubation with secondary antibodies (Thermo Fisher Scientific, Alexa-Fluor647 Goat anti Mouse, A21235) in 1% NGS/1× PBS for 1 h at RT. Finally, neurons were washed with 1× PBS (3 × 5 min) and mounted in Duolink In Situ Mounting Medium with DAPI (Sigma-Aldrich, DUO 82040). Fluorescent images were obtained with an epifluorescence microscope (Nikon Ti-E Microscope, Nikon Imaging Center at UCSF). The intensity of PLA signals, Tau1 and Tau5 immunoreactivity, and GFP fluorescence within neuronal processes were quantitated with the NIS elements software (Nikon). Since PLA signals correlated with the expression levels of mTau (Additional file 4: Figure S4C), they were normalized to colocalized Tau1 or Tau5 immunofluorescence to calculate the affinity of each mTau mutant to Fyn.

### Statistical analysis

Experimenters were blinded with respect to the genotype and treatment of cell cultures. Biological units were randomized during assays, sampling and analyses. Statistical analyses were performed with Prism (Ver. 6, GraphPad) or R (R Development Core Team, http://www.R-project.org/). Individual experiments (glutamate-induced excitotoxicity assay, live Ca^2+^ imaging, high-content imaging assay, and western blot analysis) or individual transfected neurons (PLA) were treated as independent units (n). Across experiments/plates, neurons of the same genotype that were exposed to the same experimental condition showed a reasonably consistent, dose-dependent change in alamarBlue signal or intracellular Ca^2+^ signal upon treatment with glutamate or other drugs. However, across experiments/plates we observed systematic variations in the mean and variance of neuronal responses as well as in the shapes of dose-response curves (Additional file 3: Figure S3). Therefore, we considered different wells on the same plate technical replicates and defined independent experiments as independent units (n) for statistical analysis. To analyze data obtained in multiple experiments, differences in the mean fluorescence signal intensities obtained by alamarBlue or Ca^2+^ imaging in wells containing experimentally altered cells (e.g., transduced with shTau) versus control cells (e.g., transduced with shSCR) on the same plate were computed for each dose of glutamate or another drug. The null hypothesis tested was that the median of the above differences at each dose was less than or equal to 0 across experiments/plates. To compare two different experimental conditions across different doses of glutamate, we calculated a *p*-value for an overall interaction between two conditions and dose from a one-sided, one-sample t-test performed on the mean difference in alamarBlue or intracellular Ca^2+^ signal intensities between the two experimental conditions across all doses. The above statistical approach was selected because it is non-parametric, and thus does not assume any particular systematic variation in the responses among experiments/plates or any particular parametric shape of the dose response curve. In other experiments, differences between genotypes and treatments were assessed, as appropriate, by unpaired Student’s t-test with Welch’s correction or by one-way ANOVA. The null hypothesis was rejected at *P* < 0.05. Quantitative data are presented as boxplots or means ± SEM.

## Additional files

Additional file 1: Figure S1. DNQX and TTX were bioactive. (A–C) Experiments illustrating effects of DNQX (A) and TTX (B, C). (A) A single experiment was performed to confirm that DNQX blocks AMPA-induced neurotoxicity in primary neuronal cultures. Data represent means ± SEM of technical replicates (4–6 wells per condition). WT neurons were treated with vehicle or the competitive AMPAR antagonist DNQX (20 μM) 1 h before and throughout exposure to different concentrations of AMPA. Raw AlamarBlue fluorescence measurements are shown in arbitrary units. (B, C) TTX blocked spontaneous and induced neuronal activity in acute brain slices. Slices of somatosensory cortex were prepared and pyramidal neurons were patch-clamped as described [9]. Whole-cell membrane voltage recordings were made in artificial cerebrospinal fluid (aCSF) in the absence or presence of 3 μM TTX (from the same batch used for cell culture experiments). (B) TTX prevented spontaneous neuronal activity as well as activity induced by 100 pA/200 ms current stimulations (arrowheads) or by a membrane voltage ramp (between asterisks). Scale bar indicates 20 s of recording time. (C) Representative membrane voltage responses elicited by 100 pA/200 ms current stimulations in control aCSF and after addition of 3 μM TTX. (PDF 4818 kb)

Additional file 2: Figure S2. Glutamate-induced neurotoxicity depends on NMDARs and extracellular Ca^2+^. (A, B) Experiments illustrating Ca^2+^ dependence of glutamate-induced neurotoxicity (A) and neurotoxicity caused by different glutamate receptor agonists (B). (A) One experiment was performed to confirm that reduction of extracellular Ca^2+^ counteracts glutamate-induced neurotoxicity. WT neurons were treated with Ca^2+^-containing isotonic solution or 0 mM Ca^2+^ solution 1 h prior to and throughout exposure to different concentrations of glutamate. Raw AlamarBlue fluorescence measurements are shown in arbitrary units. (B) Neurotoxicity was assessed as above in WT neuronal cultures treated with the indicated glutamate receptor agonists for 15 min at DIV 13. Data represent means ± SEM of technical replicates (8–16 wells per condition) from 1 to 2 experiments. (PDF 4803 kb)

Additional file 3: Figure S3. Variability of glutamate-induced neurotoxicity measurements across independent experiments. In each of seven independent experiments (carried out on different days), primary neurons pooled from multiple WT mice were cultured on a single 96-well plate and transduced with lentiviral vectors encoding shSCR or shTau (44–48 wells per shRNA) at the time of plating (DIV0). On DIV 13, neurons were exposed to glutamate (4–8 wells per concentration) for 15 min, followed by alamarBlue assay of neurotoxicity 24 h later. Different from the other figures, the box plots shown here represent the distribution of the raw fluorescence signals (in arbitrary units) measured in each set of wells (technical replicates) per glutamate concentration for each experimental condition (shSCR and shTau) and experiment (1–7). The lower and upper ends of the boxes represent the 25th and 75th quartile of the respective distributions. The horizontal line in each box represents the median. The ends of the whiskers terminate at the farthest points that are within 1.5 times the inter-quartile range (difference between upper and lower ends of the box). Individual dots shown in some of the panels represent outliers that fell outside the range defined by the whiskers. Note that, across experiments, neurons of the same genotype that were exposed to similar experimental conditions showed systematic variations in the mean and variance of their responses at given doses as well as in the shapes of their dose-response curves. Therefore, accounting for experiment-to-experiment variability by mean models of intensity (e.g., linear mixed effect models) and minimizing the number of dose-specific parameters to be estimated by assuming dose-response models (e.g., linear, cubic, or Hill) were not suitable for the analysis of this data. (PDF 4937 kb)

Additional file 4: Figure S4. Proximity ligation assay (PLA) to quantify association between tau and Fyn in neurons. (A) Diagram illustrating key features of the PLA assay. Neuronal cultures from *Mapt* ^−/−^ mice were transfected on DIV7 with a plasmid encoding GFP-P2A or GFP-P2A-mTau and fixed for PLA and immunostaining on DIV8. The GFP-P2A-mTau fusion protein is posttranslationally cleaved at the C-terminal side of the P2A peptide, resulting in the production of GFP-P2A and mTau at a 1:1 molar ratio. The mTau expressed was either WT or mutant (Fig. 5a). GFP signals were used to identify transfected neurons and PLA signals to measure the association between exogenous mTau and endogenous Fyn (see Methods for details). mTau immunofluorescence was used to normalize PLA signals, because mTau expression levels varied among neurons and correlated with PLA signals, as indicated in (C). (B, C) Neurons were transfected with plasmids encoding GFP-P2A, GFP-P2A-mTau^WT^, or GFP-P2A-mTau^AxxA7^ on DIV7 and analyzed by PLA and tau immunostaining on DIV8. (B) Representative photomicrographs showing levels of GFP signals (left), PLA signals (middle), and tau immunoreactivity (right) in three neurons from different culture wells. Scale bar: 50 μm. (C) Correlation between PLA signals and Tau5 immunofluorescence across individual neurons in cultures transfected with GFP-P2A-mTau^WT^ (black: R^2^ = 0.6436, *P* < 0.0001) or GFP-P2A-mTau^AxxA7^ (red: R^2^ = 0.3098, *P* < 0.0001) by linear regression analysis. Neurons transfected with GFP-P2A (gray) served as a negative control. Data points represent measurements obtained in individual neurons (mean of signals from three neurites per neuron) on two coverslips in a single experiment. (PDF 7504 kb)

## Abbreviations

AD: Alzheimer’s disease
Aβ: Amyloid-β
ASO: Anti-tau antisense oligonucleotide
shTau: Anti-tau shRNA
Ca^2+^: Calcium
EPSCs: Excitatory postsynaptic currents
hAPP: Human amyloid precursor proteins
KAR: Kainate receptor
mTau: Mouse tau
NMDAR: NMDA receptor
pY18-tau: Tau phosphorylated at tyrosine 18
PSD: Postsynaptic density
PxxP: Proline-rich motifs in tau
*Mapt*^−/−^: Tau-deficient
TTX: Tetrodotoxin
VGCC: Voltage-gated Ca^2+^ channels
