# Supplementary figures and images for: Phosphorylation of tau at Y18, but not tau-fyn binding, is required for tau to modulate NMDA receptor-dependent excitotoxicity in primary neuronal culture
Source: Mol Neurodegener. 2017 May 19;12:41. doi: 10.1186/s13024-017-0176-x (PMC5438564; doi:10.1186/s13024-017-0176-x)

# Supplementary Figure S1

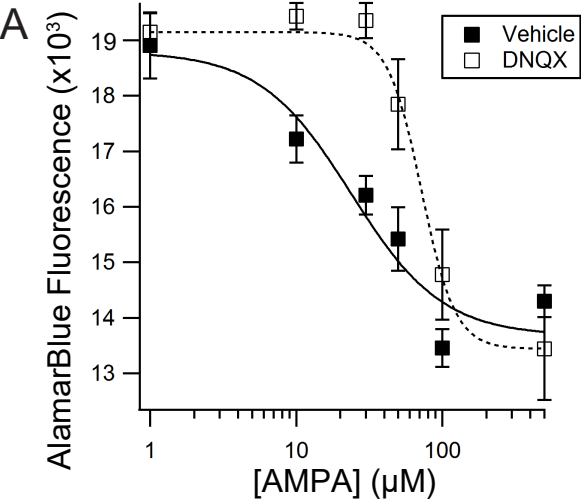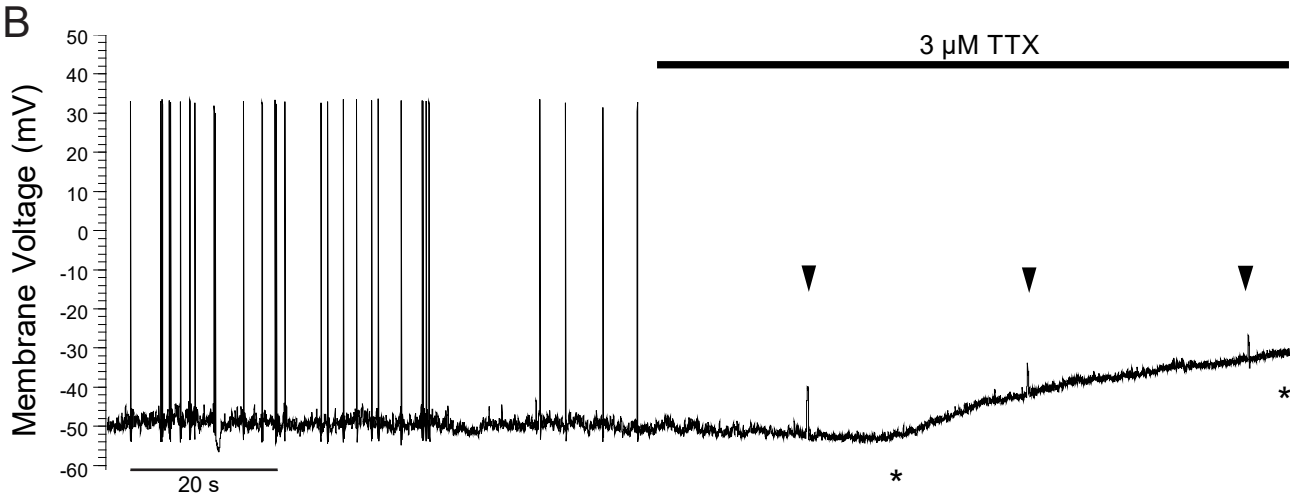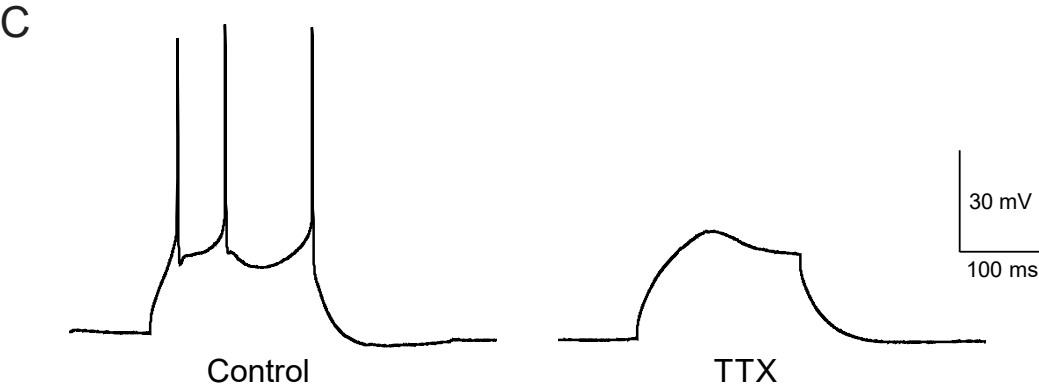

Supplement: Supplementary file 1 — DNQX and TTX were bioactive. (A–C) Experiments illustrating effects of DNQX (A) and TTX (B, C). (A) A single experiment was performed to confirm that DNQX blocks AMPA-induced neurotoxicity in primary neuronal cultures. Data represent means ± SEM of technical replicates (4–6 wells per condition). WT neurons were treated with vehicle or the competitive AMPAR antagonist DNQX (20 μM) 1 h before and throughout exposure to different concentrations of AMPA. Raw AlamarBlue fluorescence measurements are shown in arbitrary units. (B, C) TTX blocked spontaneous and induced neuronal activity in acute brain slices. Slices of somatosensory cortex were prepared and pyramidal neurons were patch-clamped as described [9]. Whole-cell membrane voltage recordings were made in artificial cerebrospinal fluid (aCSF) in the absence or presence of 3 μM TTX (from the same batch used for cell culture experiments). (B) TTX prevented spontaneous neuronal activity as well as activity induced by 100 pA/200 ms current stimulations (arrowheads) or by a membrane voltage ramp (between asterisks). Scale bar indicates 20 s of recording time. (C) Representative membrane voltage responses elicited by 100 pA/200 ms current stimulations in control aCSF and after addition of 3 μM TTX. (PDF 4818 kb) [file 13024_2017_176_MOESM1_ESM.pdf]

Supplementary Figure S2

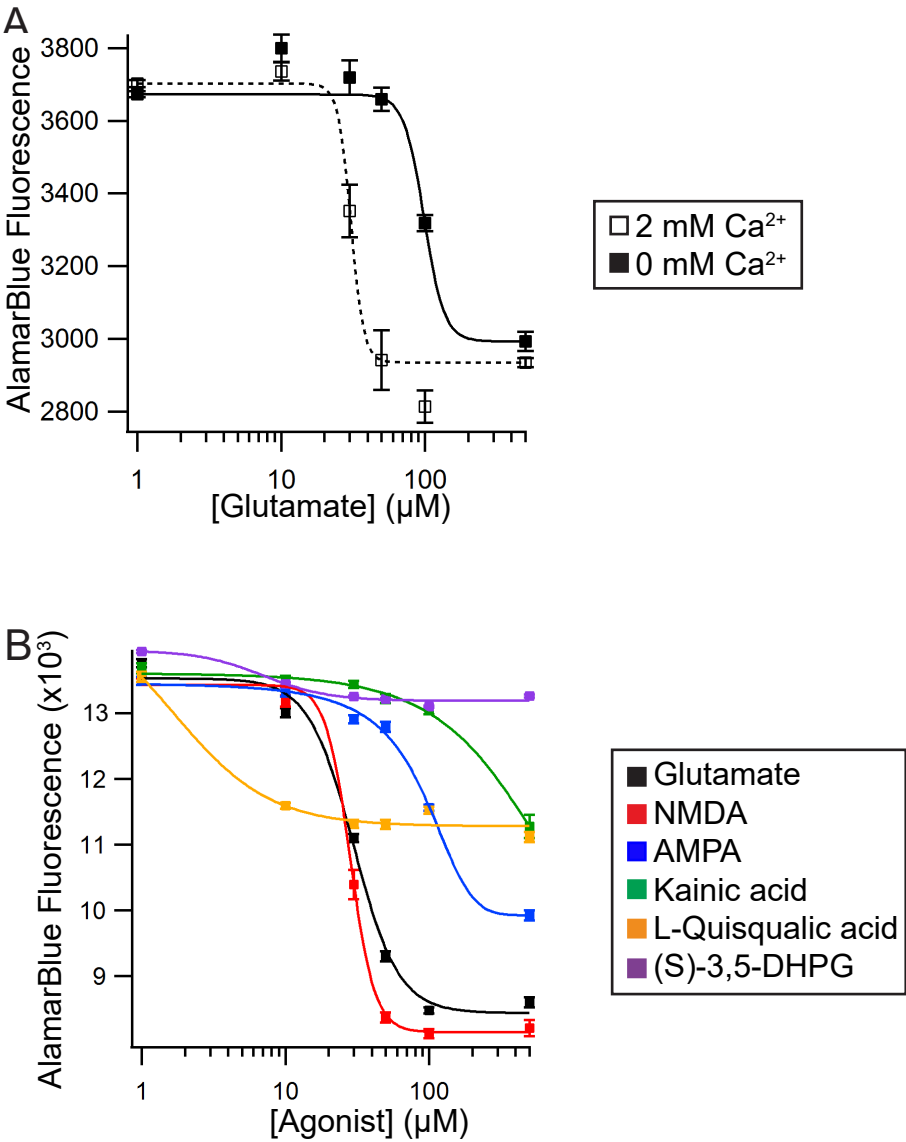

Supplement: Supplementary file 2 — Glutamate-induced neurotoxicity depends on NMDARs and extracellular Ca2+. (A, B) Experiments illustrating Ca2+ dependence of glutamate-induced neurotoxicity (A) and neurotoxicity caused by different glutamate receptor agonists (B). (A) One experiment was performed to confirm that reduction of extracellular Ca2+ counteracts glutamate-induced neurotoxicity. WT neurons were treated with Ca2+-containing isotonic solution or 0 mM Ca2+ solution 1 h prior to and throughout exposure to different concentrations of glutamate. Raw AlamarBlue fluorescence measurements are shown in arbitrary units. (B) Neurotoxicity was assessed as above in WT neuronal cultures treated with the indicated glutamate receptor agonists for 15 min at DIV 13. Data represent means ± SEM of technical replicates (8–16 wells per condition) from 1 to 2 experiments. (PDF 4803 kb) [file 13024_2017_176_MOESM2_ESM.pdf]

# Supplementary Figure S4

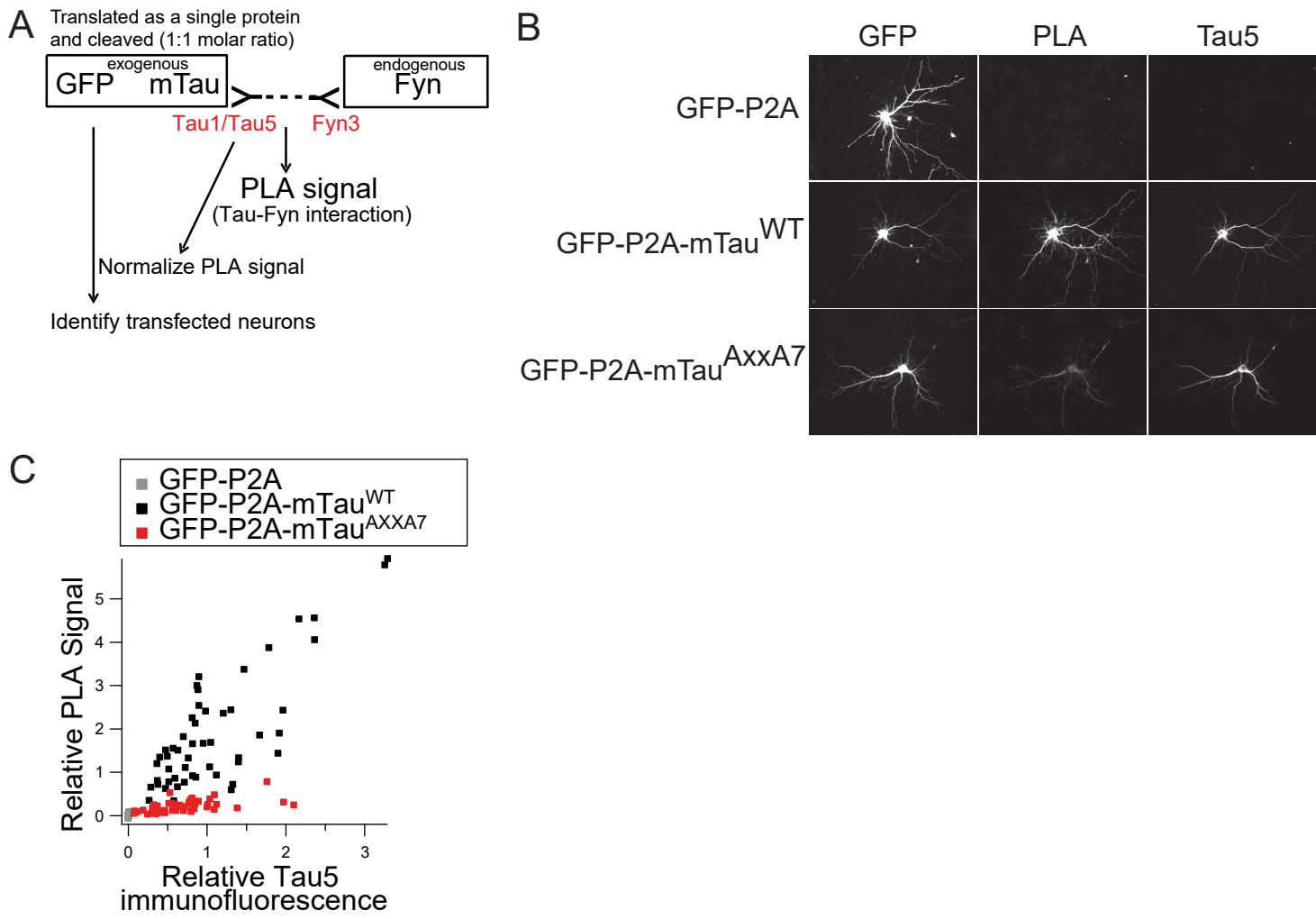

Supplement: Supplementary file 4 — Proximity ligation assay (PLA) to quantify association between tau and Fyn in neurons. (A) Diagram illustrating key features of the PLA assay. Neuronal cultures from Mapt −/− mice were transfected on DIV7 with a plasmid encoding GFP-P2A or GFP-P2A-mTau and fixed for PLA and immunostaining on DIV8. The GFP-P2A-mTau fusion protein is posttranslationally cleaved at the C-terminal side of the P2A peptide, resulting in the production of GFP-P2A and mTau at a 1:1 molar ratio. The mTau expressed was either WT or mutant (Fig. 5a). GFP signals were used to identify transfected neurons and PLA signals to measure the association between exogenous mTau and endogenous Fyn (see Methods for details). mTau immunofluorescence was used to normalize PLA signals, because mTau expression levels varied among neurons and correlated with PLA signals, as indicated in (C). (B, C) Neurons were transfected with plasmids encoding GFP-P2A, GFP-P2A-mTauWT, or GFP-P2A-mTauAxxA7 on DIV7 and analyzed by PLA and tau immunostaining on DIV8. (B) Representative photomicrographs showing levels of GFP signals (left), PLA signals (middle), and tau immunoreactivity (right) in three neurons from different culture wells. Scale bar: 50 μm. (C) Correlation between PLA signals and Tau5 immunofluorescence across individual neurons in cultures transfected with GFP-P2A-mTauWT (black: R2 = 0.6436, P < 0.0001) or GFP-P2A-mTauAxxA7 (red: R2 = 0.3098, P < 0.0001) by linear regression analysis. Neurons transfected with GFP-P2A (gray) served as a negative control. Data points represent measurements obtained in individual neurons (mean of signals from three neurites per neuron) on two coverslips in a single experiment. (PDF 7504 kb) [file 13024_2017_176_MOESM4_ESM.pdf]
