# Supplementary material for: Phosphorylation of tau at Y18, but not tau-fyn binding, is required for tau to modulate NMDA receptor-dependent excitotoxicity in primary neuronal culture
Source: Mol Neurodegener. 2017 May 19;12:41. doi: 10.1186/s13024-017-0176-x (PMC5438564; doi:10.1186/s13024-017-0176-x)

Supplementary Figure S3

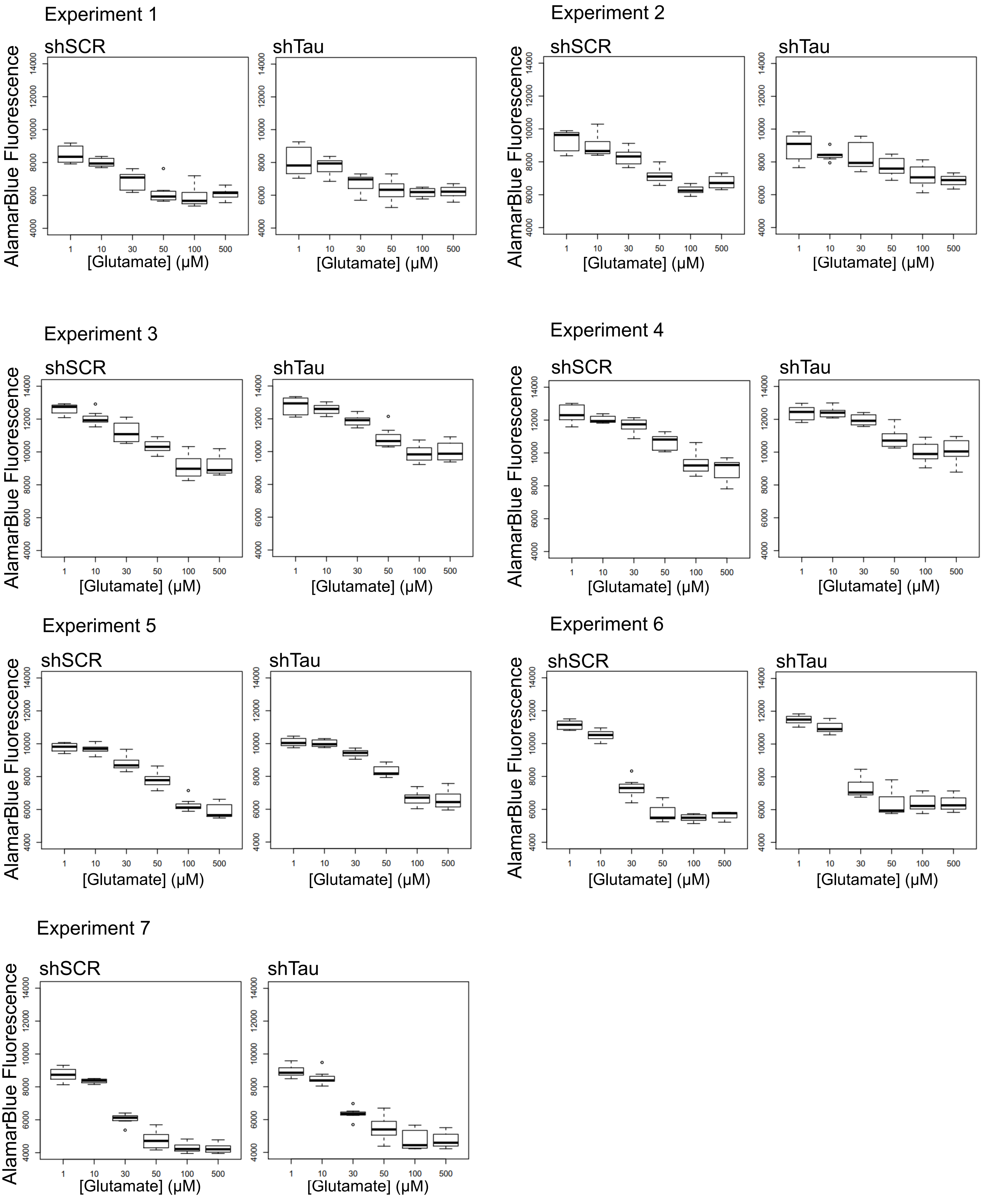

Supplement: Supplementary file 3 — Variability of glutamate-induced neurotoxicity measurements across independent experiments. In each of seven independent experiments (carried out on different days), primary neurons pooled from multiple WT mice were cultured on a single 96-well plate and transduced with lentiviral vectors encoding shSCR or shTau (44–48 wells per shRNA) at the time of plating (DIV0). On DIV 13, neurons were exposed to glutamate (4–8 wells per concentration) for 15 min, followed by alamarBlue assay of neurotoxicity 24 h later. Different from the other figures, the box plots shown here represent the distribution of the raw fluorescence signals (in arbitrary units) measured in each set of wells (technical replicates) per glutamate concentration for each experimental condition (shSCR and shTau) and experiment (1–7). The lower and upper ends of the boxes represent the 25th and 75th quartile of the respective distributions. The horizontal line in each box represents the median. The ends of the whiskers terminate at the farthest points that are within 1.5 times the inter-quartile range (difference between upper and lower ends of the box). Individual dots shown in some of the panels represent outliers that fell outside the range defined by the whiskers. Note that, across experiments, neurons of the same genotype that were exposed to similar experimental conditions showed systematic variations in the mean and variance of their responses at given doses as well as in the shapes of their dose-response curves. Therefore, accounting for experiment-to-experiment variability by mean models of intensity (e.g., linear mixed effect models) and minimizing the number of dose-specific parameters to be estimated by assuming dose-response models (e.g., linear, cubic, or Hill) were not suitable for the analysis of this data. (PDF 4937 kb) [file 13024_2017_176_MOESM3_ESM.pdf]
